# Traumatic Dental Injuries Resulting from Sports Activities; Immediate Treatment and Five Years Follow-Up: An Observational Study

**DOI:** 10.2174/1874210601812010001

**Published:** 2018-01-15

**Authors:** Enrico Spinas, Antonello Mameli, Luca Giannetti

**Affiliations:** 1 Department of Surgical Sciences, Sports Dental Research Center, University of Cagliari, Cagliari, Italy; 2 Department of Surgical Sciences, Pedodontics Division, University of Modena-Reggio, Modena, Italy

**Keywords:** Sport dentistry, Mouth-guards, Dental trauma, Recovery time, Complications, Luxation injuries

## Abstract

**Background:**

Traumatic dental injuries (TDIs) represent 18-30% of all oral pathologies and a considerable number of these are sports related. It is very important to treat sports-related injuries and prevent complications. However, very few studies investigate the most expedient therapeutic strategies for the treatment of dental trauma correlated to sports.

**Objective:**

The aim of this work was to focus on the average recovery time for different lesions, to assess adequate times for each athlete, to identify any association with complications and to investigate whether or not the use of mouth-guards interfered with a full recovery to normal health.

**Methods:**

This study involved a group of 30 athletes (15 male and 15 female) who had dental injuries of varying severity.

For the purposes of data collection, two classifications were taken into account: a classification for hard tissue trauma and another for periodontal lesions. The athletes were subdivided in “type of lesion’ groups”.They were then treated depending on their individual lesions and followed up for 5 years. A statistical analysis was carried out to study the association between recovery time, lesion types and occurrence of complications.

**Results:**

The time for recovery was different for each type of lesion and ranged from 3-5 days (only uncomplicated fractures) to 14 days (all hard-periodontal tissue traumas). The total number of recorded pulp complications amounted to 6 cases. Among 30 athletes, 20 had begun and maintained, during the five-year follow-up period, the habit of using mouth-guards when practicing their sport activities.

**Conclusions:**

Recovery time and the severity of lesions are statistically associated: the more serious the injury, the more time an athlete needs to recover and return to competitive sports events. Furthermore, recovery time and precautionary measures (mouth-guards) did not influence the onset of complications. The subjects’ habit of wearing a mouth guard continued even after the end of the therapy and follow-up periods.

## 1 INTRODUCTION

About 18-30% of all oral pathologies are traumatic injuries affecting the oral cavity and dental apparatus (TDIs) [1]; a considerable number of these are the result of sports injuries and about 25% of them involve adolescents (8-14) and young adults (15-23 anni) [2].

When such injuries occur, it is of the utmost importance that the injured subjects receive treatment as soon as possible in order to reduce and prevent complications [3].

There are very few studies in the existing literature that have addressed the question of the most expedient therapeutic strategies to adopt when treating competititve athletes [4, 5], but it is often the case that medical sport teams want the injured athlete to return to competition as soon as possible [6].

The purpose of this longitudinal study is to present a clinical protocol therapy applied to a group of 30 athletes between the ages of 8 and 20, who suffered from dento-alveolar traumas affecting the hard tissue and periodontal tissues. They were monitored for a five-year period in order to measure the healing success rate of the suffered injuries, their interferences with the athletes’ recovery time and any complications that resulted. Specifically, we studied the average time each athlete required to return to sports competitions, whether or not there were associated complications, as well as the number of athletes who started and continued the habit of using mouth-guards during their sports activities.

## 2 MATERIALS AND METHOD

This study was approved by the Institutional Review Board and informed written assent/consent was obtained from the participants and parents or guardians prior to participation.

### 2.1 Trauma Classifications

Injuries to the dento-alveolar apparatus of permanent teeth can be classified using a number of different systems [7]. The most commonly utilized system is the 1992 WHO-Andreasen system [8], used to classify injuries to the dental hard tissues and pulp as well as to the periodontium. It is subdivided into 8 descriptive classes of hard tissue injuries and 6 descriptive classes of injuries to the periodontium (Table **1**). There are, however, further classification systems that can help in collecting documentary evidence:

One of these, concerning injuries to dental hard tissues, is the Spinas Classification [9], which subdivides various types of coronal fracture into 4 classes and 3 sub-classes (Table **2**).

Both systems have been used in our study in order to provide a precise framework for the gathered data.

There are numerous recommendations and guidelines that advise on the clinical therapy to undertake according to the mouth and dental injuries a person suffers; although the most commonly used guidelines are the regularly updated ones devised by the IADT [3], there are no specific recommendations for the treatment of competition athletes.

While there are some contributions in the existing sports medicine literature [10], they are too generic with regard to the specific aims of our study. It is of the utmost importance that traumas affecting teeth and/or the periodontum of an athlete who engages in sports with a medium-to-high risk of suffering facial traumas, should be correctly diagnosed and treated so as to decide whether or not they should return to their sports activity and their recovery time. Any decision must take account of the length of time and rest required from practicing sports, the efficiency of the healing process- and therefore the success or unsuccess of long term results- and/or the potential recurrence of the consequences of an injury.

### 2.2 Study Design and Recruitment

The study involved a group of 30 athletes (15 male and 15 female) all engaged in competitive sports and aged between 8 and 20 (average age of 14). All of these young athletes had suffered a traumatic injury in the two- year period 2010-2011 while practicing their sport. They were subsequently monitored from the time of the trauma up to the year 2016, with periodic checks. The traumas were treated following the IADT protocol [3] (www.iadt-dentaltrauma.org) by the same expert practitioner, with the support of two less experienced assistants.

The data regarding age, sex, the type of sport (classified as high or medium risk) [11], number and the type of teeth involved and type of lesions are all summarized in Table **3**.

Among the injuries manifested in our group of young athletes, 15 were hard tissue injuries (tooth fracture), 10 were injuries simultaneously affecting the hard tissues (fracture) and soft tissues (luxation) while 5 were injuries affecting only the periodontal tissues (luxation), two of them had cases of dental avulsion.

Overall, 55 teeth were involved (35 hard tissue fractures and 20 involved the periodontal tissue; a total of 15 cases were teeth affected by both hard and periodontal tissue pathologies). The most affected teeth were upper central incisors (33 cases in total), upper lateral incisors (15), lower central incisors (5) and lateral teeth (2). When those injuries occurred, none of the athletes involved were wearing fixed orthodontic devices nor were they using protective mouth guards.

### 2.3 Statistical Analysis

The statistical analysis we carried out aimed to discover whether a relationship between recovery time and the type of lesions and between the onset of complications and the time needed for recovery. Due to the limited sample size, associations were sought by adapting the Fisher’s “exact” test performed using the software SAS, © SAS Institute Inc., Cary, NC 27513. The significance level was set at *P* < 0.05.

## 3 RESULTS

### 3.1 Group Subdivision

Table **3** indicates the type of oral injury suffered by each individual subject (according to the WHO’s as well the Spinas classification), the number of teeth affected, and the type of sport practiced.

This information was essential in deciding the prognosis and planned treatment and the right time for a return to sport activities.

For the sake of simplicity, we divided athletes in groups based on the type of injury and ascribed a reference code: uncomplicated crown fractures (F), complicated crown fractures with pulp involvement (FP), luxation and crown fractures (LF) and only luxation (L).

The following specific observations were noted:

5 subjects had only Enamel-dentin fractures (group F, 11 teeth, class A, B, C of the Spinas classification); 2 subjects (4 teeth) had complicated crown fractures affecting the pulp (the FP group, classes b1, c1, and d1 of the Spinas classification);

2 subjects (LF group, 3 teeth) had only dental luxation injuries and simultaneous uncomplicated crown fractures;

3 subjects (3 teeth) had luxation and classes’ c1 and d1 complicated fractures;

5 subjects (5 teeth) manifested only luxation injuries (group L), two of whom had suffered total dental avulsion;

13 subjects had mixed dental injuries; 9 of these manifested uncomplicated crown fractures (12 teeth) together with dental luxation injuries (9 teeth), making a total of 21 teeth that were placed into the mixed LF/F group;

4 subjects had complicated crown fractures (4 teeth) concurrent with uncomplicated crown fractures (4 teeth), making a total of 8 teeth that were placed into FP/F group (Table **4**).

### 3.2 Type of Trauma

The athletes in group F (5 athletes, 11 teeth) were able to return to sport competitions fairly rapidly, at most after 3-5 days from the moment the trauma occurred. None of the athletes in this group showed pulp necrosis after the 5 years study period.

In cases of class C and B fractures, the athletes were advised to use mouth protection devices such as new generation Boil and Bite (n.g. B & B) Mouth-guards (MG) [12].

The phase of modeling was undertaken under the strict control of our clinical experts using products made by QUATTROTI DENTECH, Srl (Cislago-VA-Italy), Powergard (Enso-Australia), or by Opro Shields (Herts-UK).

In the case of the FP group (2 people, 4 teeth) and the mixed F/FP group (4 athletes, 8 teeth) a similar protocol, varied according to the age of the patient given the importance of maintaining pulp vitality [13-17], was followed. The treatment given resulted in the successful reconstruction within one year of the trauma of all the teeth subjected to pulpotomies [18, 19].

In the five years follow-up period, no subject in this group manifested pulp necrosis. In all these cases the athletes were able to go back to sports competition within 5-7 days and in one case only (d1 fracture class) after 14 days (*i.e.* after the rehabilitation phase). All subjects were advised to use n.g. B & B MG protection during this period.

There was a total of five subjects in the LF group (6 teeth affected).

Another group (Gruppo LF/F) of 9 subjects comprised athletes with fractures and luxation (9 teeth) as well as other teeth manifesting crown fractures (12 teeth). In the diagnostic phase for this group, the typology of luxation was first identified and categorised [18].

Of the 15 teeth (6 in the LF group and 9 in the LF/F group) affected by both luxation and crown fractures, 8 of them were cases of subluxation, 4 were extrusive luxation and 3 teeth had intrusive luxation.

For the cases of subluxation, a splinting treatment was immediately initiated using orthodontic appliances (brackets and NIT 014/016 wire) for 14 days. For extrusive (4 teeth) and intrusive luxation (3 teeth), orthodontic repositioning treatment was initiated immediately (always within 24 hours from the trauma) [20, 21].

Repositioning therapy is generally carried out over 30/45 days and requires a further 15 days of continous verification of the obtained results.

Luxation injuries require regular monitoring over time because the affected teeth may lose vitality and develop necrosis [22].

In our cases, 4 teeth had to undergo subsequent endodontic therapy (2 with subluxation and 2 with intrusive luxation), but at the present time (5 years after the trauma), there are no further negative consequences, such as root resorption and/or ankyloses [23].

The return of these athletes to sports activities had to be gradual and was never before 7-14 days after the trauma. In any case, it was essential for them to use n.g. B & B MG, which favours a gradual adaptation to the movement and repositioning of teeth resulting from the orthodontic treatment [19, 24].

The last group of athletes with dental luxation, (group L) consisted of 5 subjects with 5 teeth affected, three of which had manifested extrusive luxation [25, 26] and two of which had manifested avulsion. The three luxated teeth were repositioned with orthodontic splints and monitored over 14 days. The three athletes in question were able to return to competition using an n.g. B & B MG.

At the end of the five year period, none of these teeth had suffered any permanent damages caused by the trauma. The undertaken therapy was more complicated for two young male athletes (aged 10 and 14) who had suffered avulsion of the two upper incisors (one central and one lateral) due to the urgent treatment required to replant the avulsed teeth. This is the chosen treatment in such cases, given the age of the patients [27, 28].

In these two particular cases, replanting was carried out a few hours after the trauma. This was classified as delayed replanting [29] with all the predictable problems associated with this procedure [30].

Having received dental replanting treatment, both these athletes made a gradual return to sports competition 14 days after the trauma, but with the obligatory use of n.g. B & B MGs. Clinical monitoring was then planned at distances of 30, 60, 90 days with further checks and x-rays repeated every 90 days for the first two years, in order to keep root resorption under clinical surveillance.

### 3.3 Statistical Results

Two points were investigated: (i) whether the type of lesion was associated with recovery time; (ii) if the time to recover was too short and risked causing complications. Regarding the first point out of 30 athletes, 5 athletes belonging to the Fracture group had recovered within 3-5 days while 25 athletes needed a recovery time longer than 5 days. Regarding the second point, the 5 athletes who recovered within 5 days did not have any complications, which were observed in athletes requiring longer recovery time.

## 4 DISCUSSION

This study has shown how traumatic dento-alveolar injuries progressed over time in a group of 30 young athletes aged between 8 and 20. Recovery time in sports after an injury to the body is a much debated subject in the relevant literature [31], but there is an evident lack of studies on the recovery time needed after traumas affecting mouth and teeth [32], and consequently a lack of protocols and procedures to follow in such cases [10].

Our observational study looked at the progression and recovery of commonly occurring dento-alveolar injuries over a period of five years when standard therapy protocols are applied [33].

In such situations it is often the case that the team hopes and expects the athlete will return soon to competition. Clearly, this should only occur if there is risk to worsen or delay recovery from the injury and/or cause the onset of complications and related problems [34].

Our study selected six groups of patients affected by various forms of injury; the groups were organised according to the gravity of the initial injury sustained (Groups F, FP, LF, L and Mixed groups LF/F, F/FP) as well as based on the number of affected teeth .

### 4.1 Type of Trauma

It was found that for the 5 subjects (11 teeth) belonging to group F, a return to sports activity (training and competition) is feasible immediately in cases of type A and B fracture, or with a delay of 3-5 days in cases of type C crown fractures.

The use of MG was not immediately necessary [35], even though their use is recommended in cases of subjects who had an as yet untreated anatomical predisposition [36].

None of the 5 subjects in this group manifested any complications or recurrences of injuries that could be related to the post-trauma treatment over the five years follow-up period.

The return to training of the 2 athletes (4 teeth in total) belonging to the FP group was possible after 7 days for one case and 14 days for the other one and subsequent intermediate therapy meant brief periods of non participation in sport (1 day on average), which never interfered with the athletes presence in competitions .

It should be pointed out that in Piccininni’s study [10], athletes with these types of injuries were expected to continue immediately with their activity, *i.e.* during a game involving professional élite athletes engaged in high level competition. In our case, the subjects were engaged in amateur sports and traumas that occurred were not examined during the game itself but always immediately afterwards (within 24 hours).

On their subsequent return to sports, the use of n.g. B & B MG was recommended. All the athletes used the recommended protection and continue to do so.

In the group of 4 athletes who had sustained mixed dental injuries (F/FP 8 teeth affected) the adopted treatment was very similar to that of the previous group.

Since these 4 subjects were also all adolescents, their recovery time was on average within 7 days, after which they began to use B & B MGs. All athletes began using protective MGs for the advised period and continue to use them.

In the LF group of 5 subjects (6 teeth) and in the LF/F group of 9 subjects (21 teeth), due to the application of orthodontic splints in all cases of extrusive and intrusive luxation, return to sports activity was always between 7 and 14 days after the trauma, and in all cases subjects used a n.g. B & B MG.

All the athletes made use of these protective devices and continued to do so for the recommended period.

The regular use of MG continued for about six months after the inital trauma and 9 subjects (out of 14 cases) have continued to use protective devices (all personalised laminated custom-made mouth guards).

For Athletes of Group L, the last group of 5 subjects (5 teeth with either luxation or avulsion), recovery time required between 7 and 14 days, with the exception of 2 cases (two teeth) that required replantation, where 14 days were needed. The continuous use of an n.g. B & B MGs was recommended in all these cases. In the cases of the replanted teeth, MG use was recommended on all further occasions, to be replaced with a laminated MG at an opportune time to ensure greater comfort and levels of performance. Three of these athletes continue to wear personalised laminated custom-made MGs.

By the end of the five years follow-up period, 20 of the athletes in the study had begun and maintained the habit of using mouth protection devices when practicing their sport. This is a highly significant information and confirms the results of previous studies [37, 38], which showed that a high percentage of subjects who suffer traumatic dental injuries use mouth guards regularly as do those who are constantly reminded to do so and who may cease to use them if not continually coaxed.

Eighteen of the 20 utilised MGs were the personalized multilayer type made using the signature technique (DREVE Dentamid- Unna-Germany) [39] and Play safe (Glidewell- Canada and Erkodent- Germany(• Erich Kopp GmbH - Pfalzgrafenweiler • Germany) [40].

The number of complications or recurrences at the end of the five years follow up period in the group who had sustained traumas, specifically 6 cases of necrosis that occurred in two subluxated, two intrused and two avulsed teeth, were no different, in percentage, to those normally expected in a group of injured subjects of the same age and sex affected by analogous typlogies of traumatic injuries that were not sports-related [41].

There were no differences in healing and recovery processes between male and female athletes.

### 4.2 Statistical Discussion

The statistical analysis of these data confirms that the two points under consideration highlight: (i) a significant association between the type of trauma and the time of recovery of the athlete's sports activity (recovery time, *P*<0.0001), which is commensurate to the trauma type, and therefore precludes traumatized athletes with complex traumas from resuming activity at a time below that established; (ii) the complications in our sample of young subjects are not statistically associated with the recovery for the purposes of competition (*P* = 0.5526) (Table **5**).

## CONCLUSION

The undertaken study examined treatment options for a group of young athletes who had all sustained a traumatic dental injury while engaged in a sports activity. The long follow-up period employed to study the group showed that an accurate classification of the injuries and their immediate treatment respecting standard protocols ensures that the subjects can return to sports competitions fairly rapidly without affecting the athletes’performance levels. What emerged in particular was the normal progress in the recovery time for the sustained injuries, with few complications or delays in rehabilitation; also, the recovery time depends on the severity of the initial trauma: the more serious the lesion, the more time an athlete needs to recover and be fit for sports activity. Indeed, the results were statistically comparable to similar trauma cases in subjects not involved in competitive sports.

It was noticeable how the numerous athletes who regularly used MGs during the recovery period did not suffer any recurrence of injuries or further traumas.

The subjects’ habit of wearing a mouth guard continued after the therapy period as well as during and after the follow up period.

There were no discernable differences in recovery time between males and females.

It will certainly be useful in the future to undertake a new study using a larger sample in order to confirm the obtained results, and to encourage the use of mouth guards as an indispensable preventive measure to avoid the risk of traumatic dental injuries, especially in adolescents.

## Figures and Tables

**Table 1:** Classification of dental and periodontal traumas (Andreasen-WHO).

**Injuries to the dental hard tissues and pulp**	• Infraction • Enamel Fracture • Enamel-dentin fracture • Enamel-dentin-pulp fracture • Crown-root fracture (uncomplicated) • Crown-root fracture (complicated) • Root fracture • Alveolar fracture
**Injuries to the periodontal Tissue**	• Concussion (shock) • Subluxation • Intrusion (central luxation) • Extrusion (peripheral luxation) • Lateral luxation • Total luxation (exarticulation)

**Table 2:** Crown fracture classification (Spinas, 2002).

Class A	Simple enamel lesions involving mesial or distal coronal angle or incisal edge
Class B	Enamel-dentin lesions involving mesial or distal coronal angle and incisal edge Subclass b1: pulp exposition
Class C	Enamel-dentin lesion involving incisal edge at least a third of the crown Subclass c1: pulp exposition
Class D	Enamel-dentin lesion involving mesial or distal coronal angle and the incisal and palatal surface with root involvement Subclass d1: pulp exposition
Class H	Fractured tooth with silent or necrotic pulp

**Table 3:** Male and female cases of teeth crown fracture (Spinas Classif.) and Luxation (WHO Classif).

**Case**	**Age**	**Sport Risk**	**Sport**	**Sex**	**Type of Fracture/Lesion**	**Teeth**	**No. of Teeth Involved**
**#1**	8	Medium	Basket	M	AL	INC C	1
**#2**	9	Medium	Basket	M	b1/b1	INC C/INC L	2
**#3**	9	Medium	Basket	M	B/c1	INC C/INC L	2
**#4**	10	Medium	Soccer	M	b1L	INC L	1
**#5**	10	Medium	Soccer	M	TL	INC C	1
**#6**	12	High	Field Hockey	M	B/A/C	INC C/INC C/INCL	3
**#7**	12	Medium	Handball	M	B/BL	INC C/INC L	2
**#8**	14	High	Field Hockey	M	B/C/AL	2 INC L/INC C	3
**#9**	14	Medium	Handball	M	CL/A	INC C/ inc c	2
**#10**	14	High	Cycling	M	TL	INC L	1
**#11**	15	Medium	Soccer	M	B/BL	INC C/INC L	2
**#12**	16	Medium	Basket	M	C/A	2INC C	2
**#13**	17	High	Rugby	M	L	INC C	1
**#14**	19	High	Rugby	M	L	inc c	1
**#15**	20	High	Field Hockey	M	A/A/AL	1 inc c/2 INC C	3
**#16**	9	Medium	Tennis	F	B/A	INC C/INC L	2
**#17**	9	Medium	Basket	F	c1L	INC C	1
**#18**	11	Medium	Handball	F	A /d1	INC C/INC L	2
**#19**	12	Medium	Basket	F	A /c1	inc c /INC C	2
**#20**	12	Medium	Basket	F	c1 /A	INC C/inc c	2
**#21**	12	High	Skating	F	b1/c1	2 INC C	2
**#22**	13	High	Field Hockey	F	C /BL	INC L/INC C	2
**#23**	14	High	Martial Art	F	C/AL	INC L/INC C	2
**#24**	14	Medium	Basket	F	L	INC C	1
**#25**	14	High	Mountain Bike	F	d1L	INC C	1
**#26**	15	High	Skating	F	CL /AL	2 INC C	2
**#27**	16	High	Cycling	F	AL/C/B	2 INC C /1 inc l	3
**#28**	17	High	Field Hockey	F	BL /A	INC L/inc l	2
**#29**	19	Medium	Basket	F	B / B	2 INC C	2
**#30**	20	Medium	Basket	F	B / C	INC C/INC L	2

Legend: Type of Lesion: A-B-C-b1-c1-d1 refer to Spinas’Classification; L = luxation; TL = Total luxation; Teeth: INC= superior incisive (C: central – L: lateral); inc = inferior incisive (c: central – l: lateral).

**Table 4:** Data regarding type of treatment, presence of complications and time to recover for each group of lesions.

Groups	**F**	**FP**	**LF**	**L**	**LF/L**	**FP/F**
** Teeth**	11	4	6	5	21	8
**Athlets**	5	2	5	5	9	4
** Type of traumas and number of teeth involved**	3A 5B 3C	2b1 2c1	2AL(e) 1CL(e) 1b1L(e) 1c1L(i) 1d1L(i)	3L(e) 2TL	LF (9): 4AL(s) 4BL(s) 1CL(i) F (12): 4A 4B 4C	F (4): 3A 1B FP (4): 3c1 1d1
** Treatment** **(IADT guidelines)**	Conservative treatment	Pulpotomy (age < 14) and conservative treatment	Orthodontic splint and conservative treatment for uncomplicated fractures; in complicated fractures also endodontic treatment (pulpotomy/pulpectomy)	3L: orthodontic splint 2TL: delayed replanting	Orthodontic splint and conservative treatment and conservative treatment for fractured teeth	Pulpotomy or pulpectomy and conservative treatment Fracture d1 needed the position of a glass fibre post
** Follow-up** **5 years**	No complications	No complications	2 intrusive luxation teeth went to pulpar necrosis and needed an endodontic therapy; after 5 years no other complications	Extrusive luxations no complications; total luxation radicular reabsorption, anchilosis and infraocclusion	2 sub-luxated teeth went to pulpar necrosis and needed an endodontic therapy; after 5 years no other complications	No complications
** Time and Precautions**	3-5 days; use ofMG is not immediately necessary	7 days; use of MG	7-14 days; use ofMG	7 days forextrusive luxations, 14 days for the totalluxation; use of MG	7-14 days; use ofMG	5-7 days; use ofMG

**Table 5:** Data studied with Fisher’s “exact” test.

**Type of Lesion and Time**				**Complications and Time**		
**Time of Recover**	**Lesions**			**Complications**		
	**F**	**Others**	**Total**	**Yes**	**No**	**Total**
**3/5 days**	5	0	5	0	5	5
**>5 days**	0	25	25	6	19	25
**Total**	5	25	30	6	24	30
**Frequency cell (1,1)**	5			0		
***P***	< 0.0001			0.5526		

**Legend:** F= uncomplicated crown fracture

## LIST OF ABBREVIATIONS

F: Only uncomplicated crown fracture;
FP: Only complicated fracture;
LF: Luxation and Fracture (same dental element);
L: Only Luxation (e = extrusive, s = subluxation, i = intrusive);
TL: Total Luxation;
LF/F: Luxation and uncomplicated fracture in different elements;
F/FP: Uncomplicated fracture and complicated fracture
MG: Mouthguard;
n.g.: New generation;
B & B: Boil and byte
